# Survival and Response Outcomes for Gastrointestinal Neuroendocrine Tumor (GEP-NETs) Patients Treated with Lutetium—177-DOTATATE in a Brazilian Reference Center: A Six-Year Follow-Up Experience

**DOI:** 10.3390/cancers15184506

**Published:** 2023-09-11

**Authors:** Zenaide Silva de Souza, Camila Bragança Xavier, Luciana Beatriz Mendes Gomes, Maria Fernanda Barbosa de Medeiros, Micelange Carvalho de Sousa, Allan Andresson Lima Pereira, José Flávio Gomes Marin, Carlos Alberto Buchpiguel, Frederico Perego Costa

**Affiliations:** 1 Oncology Center, Hospital Sírio-Libanês, Brasília 70200-730, Brazil; souzazenaide31@gmail.com (Z.S.d.S.); lucianabmg1@gmail.com (L.B.M.G.); allan.pereira.onco@gmail.com (A.A.L.P.); 2 Oncology Center, Hospital Sírio-Libanês, São Paulo 01308-050, Brazil; maria.fbmedeiros@slserv.com.br (M.F.B.d.M.); micelange22@yahoo.com.br (M.C.d.S.); jfgmarin@yahoo.com.br (J.F.G.M.); buch@usp.br (C.A.B.); fredericoperegocosta@gmail.com (F.P.C.)

**Keywords:** neuroendocrine tumors, lutetium-177, nuclear medicine

## Abstract

**Simple Summary:**

Gastroenteropancreatic neuroendocrine tumors (GEP-NETs) are the most common primary site of NETs. Peptide receptor radiation therapy with Lutetium-177-DOTATATE (PRRT) is the standard treatment for grade 1 (G1) or grade 2 (G2) midgut NETs when somatostatin analog therapy fails, but limited data exists on all-grade GEP-NETs, especially for grade 3 (G3) tumors. Here, we aimed to review the clinical-pathological and radiological characteristics of those tumors and correlate them with outcomes. That might help with how to select patients for PPRT.

**Abstract:**

Background: PRRT can be an option for all-grade GEP-NETs, but selecting patients is challenging. In this scenario, clinical-pathological and radiological characteristics, such as pre-treatment Ga-68 DOTA PET/CT, might have the potential to help. Methods: A retrospective chart review was conducted on advanced GEP-NETs treated with at least one PRRT dose. Overall survival (OS) and progression-free survival (PFS) were calculated using the Kaplan–Meier method. Krenning Score (KS), and the maximum standardized uptake value (SUVmax) were derived from the pre-treatment scans. A maximally selected rank statistics test was used for SUVmax simple cut point estimate. Results: Among 36 patients, 19 had primary pancreatic tumors. The numbers of G1, G2, and G3 tumors were 10, 18, and 7, respectively. During a median follow-up of 90.5 months, 4 patients died. Median OS was not reached for G1 and G2 tumors, and it was 30 months for G3 (*p* = 0.001). Median PFS was 23 months, with G3 showing lower PFS compared to G1 [7 versus 30 months; HR 8.41 (95%CI 2.2–31.0; *p* = 0.001)]. Conclusions: PRRT provides long-term PFS in patients with G1/G2 GEP-NETs independent of clinical characteristics and primary site. G3 has worse survival, but selected patients may experience long OS after PRRT treatment.

## 1. Introduction

Neuroendocrine neoplasms (NENs) are a rare and heterogeneous group of tumors originating from neuroendocrine cells throughout the body, although most are in the gastrointestinal tract. Once these cells can behave distinctly, the World Health Organization (WHO) improved the NENs pathologic classification in 2010, distinguishing between well-differentiated neuroendocrine tumors (NETs) and poorly differentiated neuroendocrine carcinomas (NECs) amidst all sites where these neoplasms arise. The NETs were divided into two distinct subgroups: low grade and high grade. The low-grade NETs are subdivided into two groups (G1 and G2) according to the mitotic rate and the proliferation rate (Ki-67 index). In 2019, more knowledge arose concerning the NETs and NECs molecular features. Somatic mutations in *MEN1, DAXX,* and *ATRX* are entity-defining for NETs, while NECs usually harbor *TP53* or *RB1* mutations [1], Table 1.

Gastroenteropancreatic NETs (GEP-NETs) are the most common primary site neoplasms. In our review, more data about their epidemiological incidence is needed. Zihan Xu et. al. analyzed the United States National Cancer Institute’s Surveillance, Epidemiology, and End Results (SEER) database, including 43,751 patients who received a diagnosis of GEP-NETs from 1975 to 2015. GEP-NETs incidence on different primary tumor sites (appendix, colon, small intestine, stomach, rectum, and pancreas) is rising, and the rectum is the most significant (annual percentage change [APC], 6.43; 95% CI, 5.65–7.23; *p* < 0.001). As a grade, the incidence increased on G1 GEP-NETs (APC, 18.93; 95% CI, 17.44–20.43; *p* < 0.001) [2]. Another population-based study from the National Cancer Registry and Analysis Service (NCRAS) in the UK was conducted on 63,949 tumor data between 1995 and 2018. Their analysis showed that the highest incidence considering GEP-NETs was in the small intestine (1.46 per 100,000) [3]. A Brazilian registry analysis is still needed since even the Instituto Nacional do Cancer (INCA) did not provide incidence estimates in their last publication in 2022 [4].

Regarding treatment, somatostatin analogs (SA), everolimus, and targeted therapies, including PRRT and tyrosine kinase inhibitors (TKIs), remain a cornerstone of NETs systemic treatment, while cytotoxic chemotherapy is standard for treating NECs [5]. In the phase III CLARINET trial, 204 patients were included with G1-G2 tumors to receive lanreotide 120 mg or placebo. The group intervention had PFS longer compared to the placebo in the primary analysis. The median PFS was not reached in the lanreotide group, while it was 18.0 months in the placebo group [6].

Among the other options, in the RADIANT-4 group, everolimus 10 mg once daily versus placebo demonstrated a longer median PFS (11.0 vs. 3.9 months; hazard ratio [HR], 0.48; *p* < 0.00001). However, it is important to note that this study also included lung tumors [7]. Lastly, in a study comparing sunitinib 37.5 mg per day to placebo, the sunitinib group exhibited a median PFS of 11.4 months, whereas the placebo group had a median PFS of 5.5 months (hazard ratio for progression or death, 0.42; 95% confidence interval [CI], 0.26 to 0.66; *p* < 0.001) [8].

Since the publication of the pivotal NETTER study and, more recently, the update of their results after the pre-specified long-term follow-up of 5 years, PRRT is considered standard treatment for patients with G1 or G2 GEP-NETs whose disease has progressed while on treatment with an SA [9]. Although the PFS results across these trials are not directly comparable due to inherent differences in the eligible population, in general, the results of median PFS using PRRT or other therapeutics confirm that it is an active treatment option. Thus, G3 NETs and NECs hold a poor prognosis, and their therapeutic sequencing is not standardized. Moreover, PRRT treatment outcomes of G3 NETs and NECs patients are still scant in the literature.

Additionally, it is worth mentioning that PRRT was an effective therapeutic option for GEP-NETs with Krenning Scores (KS) of 2, 3, or 4 at Octreoscan [8]. This stratification of patients is based on the presence and the degree of radiopharmaceutical uptake on somatostatin receptor scintigraphy or PET/CT examinations, with scores varying from zero (no tumor uptake) to 4 (very intense tumor uptake), which by the end reflects the level of somatostatin expression in the detected lesions. Over the last few years, somatostatin receptor scintigraphy has become less used because of the advantages of Ga-68 DOTA(TATE/TOC/NOC) PET/CT scan (Ga-68 DOTA PET/CT), which include higher resolution and sensitivity leading to superior accuracy, faster acquisition times, and lower radiation exposure [10].

The purpose of this study was to describe the six-year experience of a Brazilian reference center treating all-grade NETs with PRRT, with special emphasis on the clinical outcomes, nuclear medicine response assessment, and treatment efficacy among the subgroup of G3 NETs and NECs.

## 2. Materials and Methods

**Figure 1:**
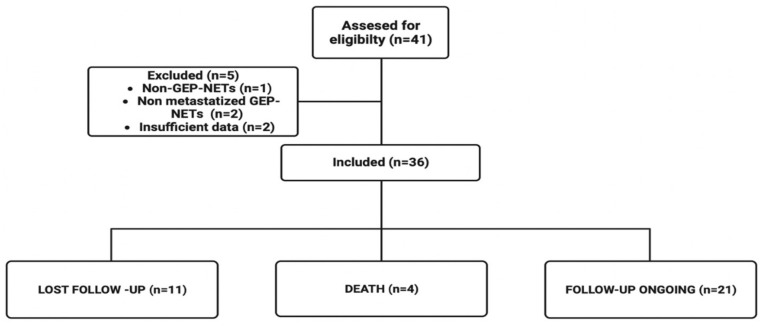
Study flowchart.

We retrospectively included patients with the following eligibility criteria: adults (age ≥ 18 years) who had advanced GEP-NETs and who were assigned to PRRT treatment (an activity of 100–200 mCi of Lu-177 DOTATATE) at the Nuclear Medicine Department of Hospital Sírio-Libanês from April 2016 to July 2022 and received at least one cycle. Exclusion criteria were primary tumor sites other than GEP, insufficient data in the medical record, or primary NECs. All the data collected was retrieved from online medical charts after ethics committee approval. A flowchart of the study design is available in Figure 1.

Patient clinical data was extracted by four independent reviewers (Z.S.S., L.B.M.G., M.F.B.M. and M.C.S.), and discordant cases were analyzed by a fifth reviewer (C.B.X.). Then, the following features were collected from the medical charts: birth date; gender; Eastern Cooperative Oncology Group (ECOG) status at first and last appointments; primary tumor site (pancreatic or non-pancreatic), tumor grade (G1, G2 or G3); the number of metastasis sites (1 or ≥2 sites); the presence of liver metastasis, the date of PRRT treatment and whether it was delivered concomitant with octreotide or not; the number of previous/post-PRRT local or systemic treatments. Somatic or germline next-generation sequence (NGS) data were collected when available (*n* = 2). Adverse events related to treatment were categorized based on Common Toxicity Criteria (CTCAE) version 5.0. Nuclear imaging results were obtained by one independent and experienced (>10 years) board-certified reader (J.F.G.M.). The KS, the maximum standardized uptake value among all lesions in each patient (SUV_max_), the lowest maximum standardized uptake value among all lesions in each patient (lowest SUV_max_), the intraindividual range of SUVmax among all lesions (given by the formula SUV_max_—lowest SUV_max_), the intraindividual lesion heterogeneity coefficient (HC, given by the formula (SUV_max_—lowest SUV_max_)/SUV_max_, where 1 is the most heterogeneous disease and 0 is the least heterogeneous—or most homogeneous disease), the mean standardized uptake value SUV (SUV_mean_), the whole body molecular tumor volume (mTV), and the whole body total lesion somatostatin expression (TLSE) were calculated from the baseline Ga-68 DOTA PET/CT in the 35 patients who underwent the scan previously to the PRRT therapy. If post-PRRT Ga-68 DOTA PET/CT or 18-Fluorodeoxyglucose PET/CT (18-F FDG PET/CT) were available during the clinical follow-up, the same features were registered for both studies.

The primary clinical outcomes were overall survival (OS) and post-PRRT progression-free survival (PFS). They were calculated using the Kaplan–Meier (KM) method and reported as a median with a 95% confidence interval (95% CI). Patients were stratified by tumor grade (G1, G2, G3), primary tumor site (pancreatic versus non-pancreatic), number of metastasis sites (1 site versus ≥2 sites), presence of liver metastasis (yes versus no), number of previous systemic treatments (1 versus ≥2), and previous local treatments (yes versus no). Cox regression analysis was performed for PFS adjusted by the above-mentioned stratification features. Multivariate analysis results were reported as hazard ratio (HR) with 95% CI. Finally, PFS was calculated as pre-treatment Ga-68 DOTA PET/CT KS, SUV_max_, lowest SUV_max_, range of SUV_max_, HC, SUV_mean_, mTV, and TLSE. Using the R 4.1.3 software, the maximally selected rank statistics (MSRS) test was used to calculate a simple cut-point estimate for all the previous features listed with the exception of KS, which is already a categorical measure. PFS was calculated using the KM method. For all statistical analyses, a significance level of *p* ≤ 0.05 was adopted for statistical significance.

## 3. Results

### 3.1. Patients’ Characteristics

A total of 36 patients were included. The median follow-up was 90.5 months (range 16–122). The cohort’s median age was 51 years (range 33–80). The tumor distribution was 19 (52.8%) primary pancreatic and 17 (47.2%) non-pancreatic. Of all samples, 7 (20%) were classified based on Ki67% as G3, 18 (51.4%) as G2, and 10 (47.2%) as G1. Twenty-two (22) patients (61.1%) had only one site of metastasis. All patients with Ga-68 DOTA PET/CT performed previously to PRRT showed high tumor uptake, predominantly >liver uptake (Krenning scale ≥ 3). Regarding molecular features, none of the patients had somatic NGS, and two had germline NGS, but both disclosed variants of uncertain meaning (VUS) at the TSC2, POLD1, and RECQL4 genes. Patients’ characteristics are summarized in Table 2.

### 3.2. Survival Outcomes

**Figure 2:**
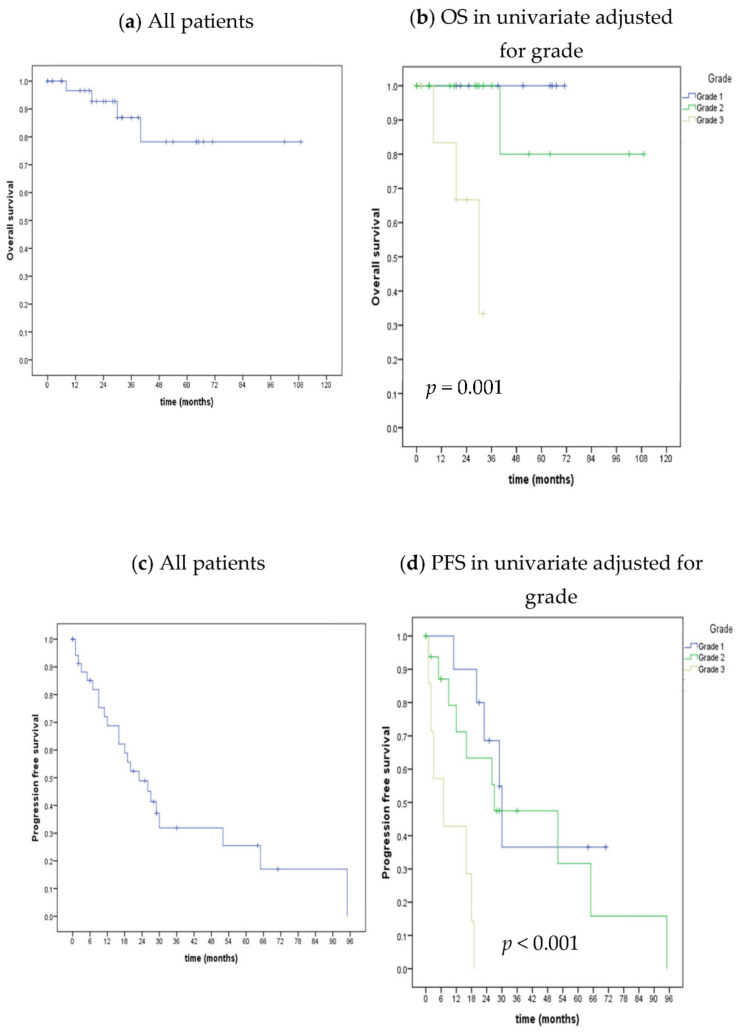
Kaplan–Meier method for OS and PFS including all patients and adjusted for grade.

Considering the 36 patients evaluated, the median OS in all groups was not reached. At 24 and 36 months, 92.7% (95% CI 73.7—98.1) and 86.9% (95% CI 63.5–95.7) of patients were alive, respectively. The median OS was not reached for G1 and G2 tumors, while it was 30 months for G3 tumors (*p* = 0.001), Figure 2a,b.

At the time of the data cutoff, 34 patients were eligible for PFS analysis. The sample median PFS was 23 months (95% CI 30.5–64.9). In the univariate analyses, the median PFS was inversely proportional to the tumor grade as follows: 30 months (range 64.2–69.2) for G1, 27 months (range 20.0–70.8) for G2, and 7 months (range 9.7–73.4) for G3, Figure 2c,d. The latest subgroup was associated with lower PFS in both univariate and multivariate analysis compared to G1 [7 versus 30 months; HR 8.41 (95%CI 2.2–31.0; *p* = 0.001)]. In the univariate analysis, neither pancreatic versus non-pancreatic primary site (*p* = 0.493), one versus two or more sites of metastasis (*p* = 0.407), one versus two or more previous systemic treatments (*p* = 0.486), or the presence versus the absence of secondary liver lesions (*p* = 0.848) reached statistical correlation with PFS. Patients who did not receive previous local treatments exhibited no PFS benefit (*p* = 0.117).

Likewise, in the multivariate analyses, primary site (hazard ratio 1.316; 95% confidence interval [(CI)], 0.522 to 3.32; *p* = 0.561), metastasis sites (HR 1.29; 95% CI, 0.514 to 3.235; *p* = 0.587), number of previous systemic treatments (HR 1.386; 95% CI, 0.5 to 3.843; *p*= 0.530), and presence of secondary liver lesions (HR 2.608; 95% CI, 0.316 to21.526; *p*= 0.373) did not reach statistical correlation with PFS, Table 3.

### 3.3. Toxicity

Overall, PRRT was well tolerated. In total, 12 (33.3%) patients disclosed toxicity. The most frequent were fatigue (*n* = 4) followed by isolated platelet count decreased (*n* = 2) and hypocellular bone marrow (*n* = 2). Other events registered and related to PRRT included abdominal pain (*n* = 1), flushing *(n* = 1), nausea (*n* = 2), arthralgia (*n* = 1), alopecia (*n* = 1), face edema (*n* = 1), hypertension (*n* = 1). Flushing and face edema developed in one patient during the first cycle of PRRT with an activity of 200 mCi. The flushing improved with treatment interruption. Afterward, the infusion was resumed at a lower rate without other symptoms. A few hours later, the patient experienced sudden flushing associated with eyelid and lip edema. The symptoms resolved after administering an antihistamine and hydrocortisone, raising the possibility of a probable acute allergic reaction. The following planned PRRT infusions were suspended.

**Figure 3:**
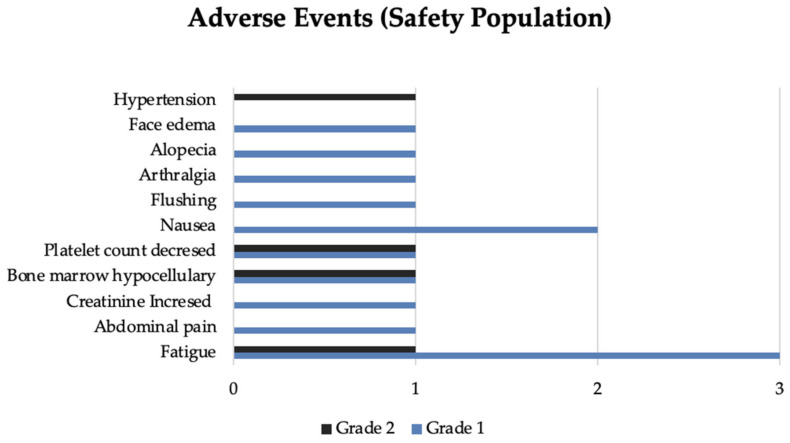
Adverse events graded using CTCAE (version 5.0).

Seventeen patients did not experience PRRT toxicity, and 6 had unavailable safety data. No grade 3 or 4 toxicities were reported. Only four patients reported more than one toxicity related to PRRT over the cycles. Adverse events incidence is represented in Figure 3.

### 3.4. Therapeutic Predictive Factors Related to Nuclear Imaging Findings

Thirty-five patients (97.2%) had pre-treatment Ga-68 DOTA PET/CT. All patients had a KS of at least 3, with 28 of them having a KS of 4. There was no significant difference in median progression-free survival (PFS) between patients with KS 3 and KS 4 (19.8 versus 28.0 months; *p* = 0.17).

**Figure 4:**
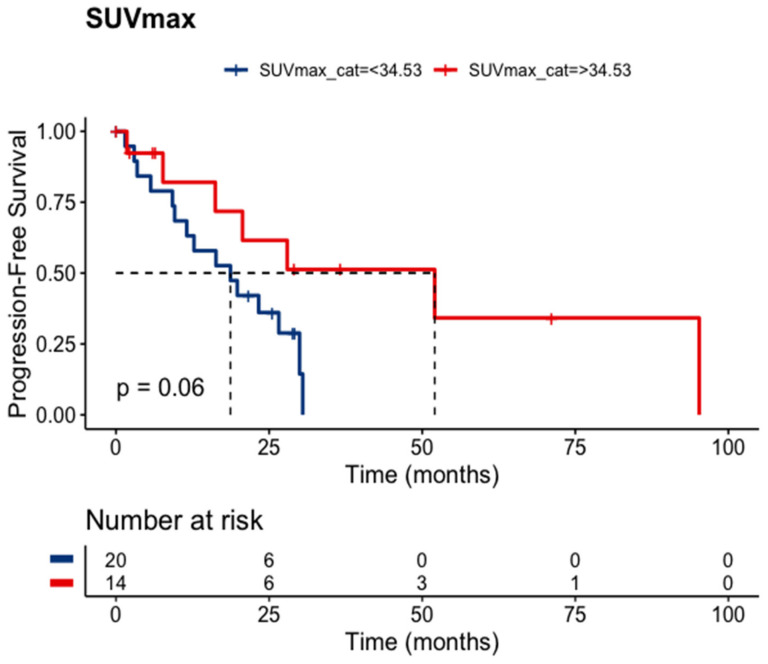
PFS according to SUV_max_ (*n* = 34).

SUV_max_ was available for 34 patients. The estimated (or calculated) SUV_max_ cut point was 34.53. Patients holding an SUV_max_ above this value exhibited a statistical trend toward a longer median PFS (52.0 versus 18.7 months; *p* = 0.06), Figure 4. Unexpectedly, the lowest SUV_max_ (cut point 8.08) holds an inverse relationship with the median PFS (*p* = 0.0022). These findings suggest that certain nuclear features may be predictive of treatment response in this patient population.

All baseline Ga-68 DOTA PET/CT features are shown in Supplementary Table S1.

**Figure 5:**
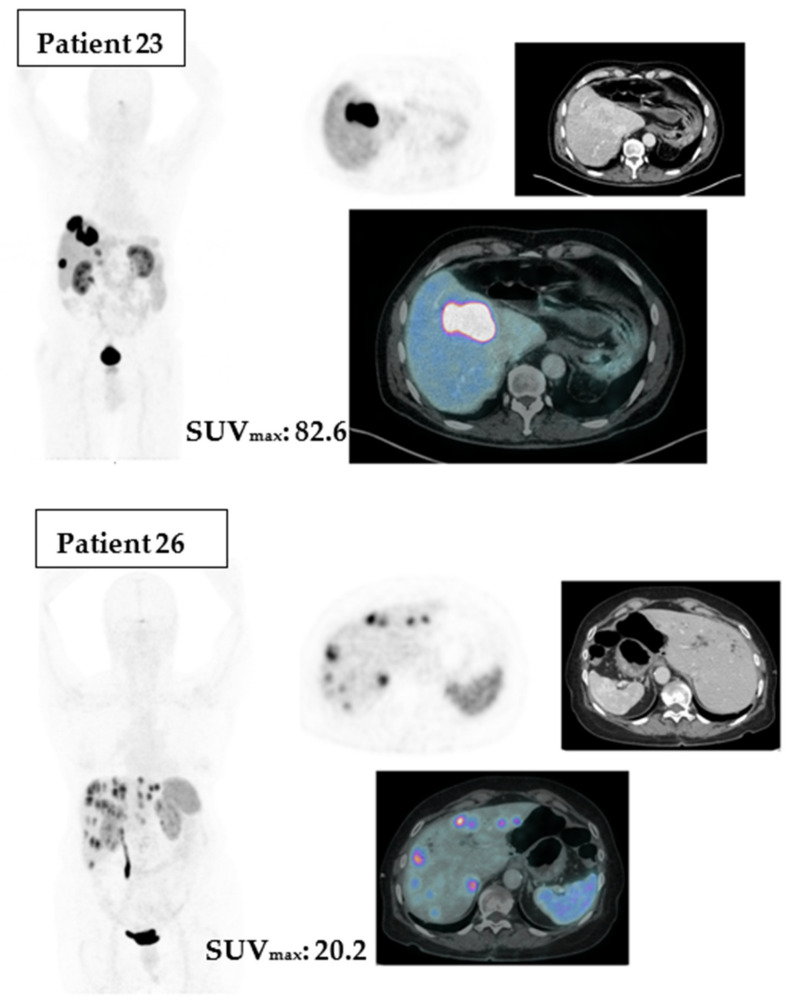
Patients of our cohort with different SUVmax.

To illustrate the differences between Ga-68 DOTA PET/CT SUVmax above or under the cut point, we point out two cases in Figure 5.

## 4. Discussion

In an era characterized by discoveries around cancer treatments, GEP-NETs still have much to reveal. This study aimed to improve our knowledge about GEP-NETs treated in our institution, especially under two main aspects. The first one was to investigate the behavior and outcomes of each subgroup (G1, G2, G3) after PRRT, and the second was to investigate potential predictive factors of Ga-68 DOTA PET/CT findings to better select the good and bad responders for PRRT.

This retrospective study included 18 (51.4%) G1, 18 (51.4%) G2, and 7 (20%) G3 GEP-NETs. After a median follow-up of 90.5 months, only 4 (11.1%) patients died, with a median OS of not reached for G1 and G2 tumors and 30 months for G3 tumors (*p* = 0.001). Median PFS was 23 months (95% CI 30.5–64.9), and all patients had at least a Krenning score of 3, with no grade 3 or 4 toxicity observed.

Our real-world data is particularly useful for analyzing rare diseases, such as high-grade tumors GEP-NETs, excluded from the randomized trials available. In the randomized NETTER-1 trial, 116 GEP-NETs patients that received PRRT were classified as low-grade (G1; *n* = 76 (66%)), intermediate grade (G2; *n* = 40 (35%)) and high-grade (G3, Ki67 >20%; *n* = 0 (0%)). Overall, 9% of them had KS 2, and in our sample, none. It is important to highlight that image selection was performed by somatostatin receptor scintigraphy in that trial and by PET/CT in our study. Mainly for small lesions, this difference could be explained by the higher spatial resolution and sensitivity of PET/CT. Their results revealed a median OS of 48 months and a median PFS not reached.

Our study population was distinct from those usually included in phase III trials, which are often limited to North American and European regions. The Brazilian population involved in this study comprises individuals with diverse ethnic backgrounds and genetic makeups. The study was conducted in a tertiary private practice center, allowing the attending physician to determine the optimal course of treatment for each patient. Additionally, the Nuclear Medicine Department and the Oncology Center were in close proximity, facilitating in-person discussions about each case, which was a significant advantage.

Furthermore, our retrospective study is limited by a small cohort size and the heterogeneity of previous PRRT, local, and systemic treatments. These factors reduce our ability to conduct a comprehensive analysis of the objective response rate and limit our findings regarding the occurrence of severe adverse events. To date, another retrospective cohort study at 12 centers has assessed the efficacy and toxicity of PRRT in 149 patients with GEP-NENs, including 58 patients with NETs G3. The study found that 42% of NETs G3 patients had a partial response, 39% had stable disease, and 7% had progressive disease. Notably, no complete responses were observed. The study also reported acute and long-term adverse events, with acute adverse events occurring in 13% of patients, most frequently affecting the hematological and renal systems. Long-term adverse events occurred in 10% of patients with the same organs affected [11].

A multicenter, randomized, phase 2, ongoing trial, OCLURANDOM, assesses PRRT versus sunitinib in pancreatic NETs. Forty-one patients on the PPRT arm were included; 33 (81%) were G2–G3, and 17 (42%) had received >2 treatment lines. Initial results showed 80% of patients without disease progression in 12 months in the PRRT arm. The median PFS estimated is 20.7 (17.2–23.7) months for PRRT versus 11.0 (8.8–12.4) months with sunitinib [12]. These preliminary results on pancreatic NETs go toward our hypothesis. However, the primary tumor site may not interfere with outcomes such as PFS after PRRT treatment.

Due to a scarcity of predictors for PRRT treatment response, we turned back to pre-treatment Ga-68 DOTA PET/CT registered; after statistical analyses of their parameters, SUVmax was the most relevant of them. The cutoff of 34.53 established by the MSRS method on our sample showed that an SUVmax > 34.53 exhibited a statistical trend toward a longer PFS (*p* = 0.06). Our molecular imaging analyses also have limitations to disclose. Ga-68 DOTA PET/CT images were acquired after different injection periods, radiopharmaceuticals (Ga-68 DOTATOC and DOTATATE), and machines. Nevertheless, the SUVmax shows a promising predictor awaiting further data in prospective studies.

## 5. Conclusions

In addition to the known benefit in G1 and G2 midgut NETs, PRRT provides better survival independent of the primary site and for G3 for selected patients. The identification of these patients with predictive biomarkers or clinical characteristics, as well as optimal treatment sequencing, remains complex and should be pursued. Ga-68 DOTA PET/CT images are nowadays underused, and it has the potential to be a predictor for PRRT, which was highlighted in our manuscript.

## Figures and Tables

**Table 1:** NENs pathologic classification and somatic mutations.

	Differentiation Pattern	Mitotic Rate	Ki67 Index (%)	Somatic Mutations
G1	well-differentiated	<2	<3	*MEN1*, *DAXX*, *ATRX*
G2	well-differentiated	2–20	3–20	
G3	well-differentiated	>20	>20	
NEC	poorly-differentiated	>20	>20	*TP53*, *RB1*

G1 = grade 1; G2 = grade 2; G3 = grade 3.

**Table 2:** Baseline characteristics of 36 patients with GEP-NETs receiving PRRT.

**Age—years, mean**	**52.1**
**Follow-up—yr, mean**	6.3
**Sex—no. (%)** Male Female	23 (63.88) 13 (36.11)
**Median time since diagnosis—yr.**	5.71
**Time since diagnosis—no.(%).** ≤3 years >3 years	9 (25) 27 (75)
**ECOG (First appointment)—no.(%)** 0–1 ≥2 Not available	12 (33.33) 0 24 (66.66)
**Primary tumor site—no.(%)** Pancreatic Non-pancreatic	19 (52.8) 17 (47.2)
**Primary site Ki67 index—no. (%)** ≤2% 3–20% >20%	10 (28.6) 18 (51.4) 7 (20)
**Number of sites of metastasis—no.(%)** 1 ≥2	22 (61.11) 14 (38.88)
**Presence of secondary liver lesions—no.(%)** Yes No	31 (86.11) 5 (13.88)
**Number of previous systemic therapies—no. (%)** 0 1 ≥2	2 (5.55) 10 (27.77) 24 (66.66)
**Previous local therapies—no. (%)** Yes No	21 (58.33) 15 (41.66)
**Ga-68 DOTA PET-CT Krenning score—no. (%)** Grade 3 Grade 4 Not available	7 (19.44) 28 (77.77) 1 (2.77)

**Table 3:** Multivariate Cox regression analysis of predictors for PFS.

Covariate	Hazard Ratio (95% CI) *p*-Value	
G1 G2 G3	1 1.449 (0.477–4.399) 8.418 (2.283–31.034)	0.513 0.001
Primary site (pancreatic vs. non-pancreatic)	1.316 (0.522–3.320)	0.561
Number of sites of metastasis (1 vs. > 2)	1.290 (0.514–3.235)	0.587
Presence of secondary liver lesions (Yes)	2.608 (0.316–21.526)	0.373
Number of previous systemic therapies (>2)	1.386 (0.500–3.843)	0.53
Previous local therapies (Yes)	2.438 (0.919–6.465)	0.073

## Data Availability

Not applicable.

## Supplementary Materials

The following supporting information can be downloaded at: https://www.mdpi.com/article/10.3390/cancers15184506/s1. Supplementary Table S1. Ga-68 DOTA PET/CT features.
