# Supplementary material for: Survival and Response Outcomes for Gastrointestinal Neuroendocrine Tumor (GEP-NETs) Patients Treated with Lutetium—177-DOTATATE in a Brazilian Reference Center: A Six-Year Follow-Up Experience
Source: Cancers (Basel). 2023 Sep 11;15(18):4506. doi: 10.3390/cancers15184506 (PMC10526125; doi:10.3390/cancers15184506)
Supplement: Supplementary file 1 [file cancers-15-04506-s001.zip › cancers-2484312-supplementary.pdf]

Supplement

Supplementary Table S1. Ga-68 DOTA PET/CT features

Patient	KS	SUVmax	Lowest SUV max	Range SUVmax	CH	SUV mean	mTV	TLSE	Number Cycles LU 177
**1**	4	21.2	5.98	15.22	0.72	9.55	457.58	4367.8	1
**2**	4	26	12.79	13.21	0.51	11.76	525.85	6181.45	4
**3**	3	14.3	1.86	12.44	0.87	2.33	13.57	31.57	4
**4**	4	39.2	1.72	37.48	0.96	9.08	1191.37	10817.01	4
**5**	-	-	-	-	-	-	-	-	4
**6**	3	16.13	4.96	11.17	0.69	9	185.2	1667.47	3
**7**	4	36.6	32.0	4.06	30.54	0.83	7.27	65.88	4
**8**	4	34.7	4,19	30.51	0.88	3.89	26.13	101.72	4
**9**	3	21.4	3.74	17.66	0.83	7.3	349.91	2555.83	4
**10**	4	58.3	9.27	49.03	0.84	14.49	84.43	1223.07	6
**11**	4	25.1	15.83	9.27	0.37	12.2	739	9016.34	4
**12**	4	35.1	4.2	30.9	0.88	10.88	233.95	2544.84	5
**13**	3	17.8	3.64	14.16	0.8	6.71	133.11	893.51	4
**14**	4	25.3	18.37	6.93	0.27	12.63	24.33	307.25	4
**15**	4	28	7.23	20.77	0.74	6.19	25.91	160.37	4
**16**	4	21.1	6.8	14,3	0.68	7.23	19.05	137.65	4
**17**	4	-	-	-	-	-	-	-	6
**18**	4	23.4	2.11	21.29	0.91	10.58	298.4	3158.41	3
**19**	4	20.75	10.0	10.75	0.52	11.78	1003.05	11819	4
**20**	4	66.8	7.31	59.49	0.89	9.25	66.37	614.23	4
**21**	4	60.1	3.28	56.82	0.95	16.76	41.41	694.05	4
**22**	4	29.4	3.6	25.8	0.88	10.85	1771.02	19209	4
**23**	4	82.6	8.08	74.52	0.9	31.74	69.09	2193.03	4
**24**	4	22.8	3.99	18.81	0.83	5.71	9.2	52.56	3
**25**	4	20.3	7.13	13.17	0.65	6.54	33.04	215.95	4
**26**	4	20.2	6.52	13.68	0.68	8	86.3	690.48	4
**27**	4	48	14.36	33.64	0.7	13.16	23.38	307.57	3
**28**	4	29.9	8.35	21.55	0.72	9.77	38.44	375.75	2
**29**	4	34.9	8.47	26.43	0.76	22.03	48.9	1077.31	3
**30**	4	66.2	8.64	57.56	0.87	20.13	43.8	881.75	4
**31**	4	51.2	8.87	42.33	0.83	14.8	2063.94	30554.4	4

**32**	3	41.3	4.08	37.22	0.9	11.89	221.04	2627.68	1
**33**	3	34.9	4.5	30.4	0.87	17.21	37.16	639.5	2
**34**	4	34.53	-	-	-	-	-	-	1
**35**	4	53.3	5.61	47.69	0.89	12.09	48.06	580.81	1
**36**	3	33	6.67	19	12.33	4.71	103.25	486.07	1

Krenning scale (KS),maximum standardized uptake value among all lesions in each patient (SUV_{max}), lowest maximum standardized uptake value among all lesions in each patient (lowest SUV_{max}), range of SUVmax, heterogeneity coefficient (HC), mean standardized uptake value SUV (SUV_{mean}), whole body molecular tumor volume (mTV), whole body total lesion somatostatin expression (TLSE).
